# Pathogenic variants screening in seventeen candidate genes on 2p15 for association with ankylosing spondylitis in a Han Chinese population

**DOI:** 10.1371/journal.pone.0177080

**Published:** 2017-05-11

**Authors:** Mengmeng Wang, Lihong Xin, Guoqi Cai, Xu Zhang, Xiao Yang, Xiaona Li, Qing Xia, Li Wang, Shengqian Xu, Jianhua Xu, Zongwen Shuai, Changhai Ding, Faming Pan

**Affiliations:** 1 Department of Epidemiology and Biostatistics, School of Public Health, Anhui Medical University, Hefei, Anhui, China; 2 Department of Rheumatism and Immunity, the First Affiliated Hospital of Anhui Medical University, Hefei, Anhui, China; 3 Menzies Institute for Medical Research, University of Tasmania, Hobart, Tasmania, Australia; Peking University First Hospital, CHINA

## Abstract

**Objectives:**

Previous studies have found the association between rs10865331 in 2p15 area and ankylosing spondylitis (AS). This study aimed to identify additional functional genetic variants in 2p15 region associated with AS susceptibility.

**Methods:**

We used next generation sequencing (NGS) in 100 AS cases and 100 healthy controls to screen AS susceptible genetic variants, and validated these variants in 620 cases and 620 controls by using imLDR^TM^ technique for single nucleotide polymorphism (SNP) genotyping.

**Results:**

Totally, we identified 12 SNPs that might confer susceptibility to AS. Of those SNPs, three (rs14170, rs2123111 and rs1729674) were nominally associated (*P*<0.05) with AS, but were no longer statistically significant after Bonferroni correction. After stratified by gender, another two SNPs (rs11428092 and rs10208769 in *USP34*) were associated with AS in males but not females, though this was not statistically significant after Bonferroni correction. In addition, rs1729674, rs14170, rs2123111 and rs10208769 were in strong linkage disequilibrium (LD) and were further enrolled in haplotype analysis. A novel haplotype TAGA was found to be associated with a decreased risk of AS (odds ratio (OR) (95% confidence interval (CI)) = 0.832 (0.705–0.982)). Beyond that, we also demonstrated a strong relationship between rs10865331 and AS susceptibility (OR (95% CI) = 1.303(1.111–1.526)).

**Conclusions:**

rs14170 and rs2123111 in*USP34* and rs1729674 in *C2orf74* may be associated with AS susceptibility in Han Chinese population. *USP34* and *C2orf74* in 2p15 region may be AS novel susceptibility genes.

## Introduction

Ankylosing spondylitis (AS) is a chronic inflammatory autoimmune disease which mainly affects sacroiliac joints and axial skeleton, causing characteristic inflammatory back pain and stiffness [1]. It affects 0.24% of Chinese populations [2],0.3% -0.5% of white Europeans, and 0.1% -1.4% of the global population [3], with a high prevalence in male youths [4]. Although the precise pathogenesis of AS has not been well understood, it is now widely accepted that genetic factors play an indispensable role in the development of AS. Several studies [5, 6] have confirmed that human leukocyte antigen B27 (HLA-B27) gene was strongly associated with the susceptibility of AS, but it only accounts for 16% of the total genetic risk for the disease, suggesting that a large amount of genetic factors in AS manifestation beyond MHC region are yet to be determined [7]. Genome-wide association studies (GWASs) [8] in European population has reported an association between the gene desert area in chromosome 2p15 and AS susceptibility, which has been replicated in a number of studies in Europeans [9] and Asians [10–13]. In addition, Brown [14] reported that the contribution rate of 2p15 area on AS genetic degrees was 0.54%, which was greater than that of another two AS susceptibility genes, *IL23R* (0.31%) and *ERAP1* (0.31%)[8]. Moreover, 2p15 has been a susceptible area with the highest genetic contribution on AS, in addition to HLA-B27.

2p15region includes the desert area and a large number of genes. At present, only one desert area susceptibility locus has been identified, while the remaining genetic information in 2p15 region is not clear. Also, the function of the positive locus rs10865331 identified by GWAS, which is located in the gene desert, is unknown. However, this cannot fully explain the high genetic contribution of this region on AS. Therefore, we hypothesized that there might be other susceptibility genes in addition to the existing susceptibility loci in the gene desert area on 2p15.

To further assess the role of 2p15 in the development of AS in Han Chinese population, we carried out a two-stage study by using a) next generation sequencing (NGS) for selected seventeen candidate genes in the discovery phase, and b) imLDR^TM^ technique in the subsequently promising SNP genotyping validation phase.

## Methods

### Study subjects

The study was approved by the ethics committees of Anhui Medical University (Hefei, China). All participants provided their written consents after being informed about the details of the study. We performed a two-step case-control study. The first stage included 100 AS patients and 100 healthy controls. Additional 620 unrelated AS cases and 620 controls were recruited in the second stage. All AS patients were from the Department of Rheumatology and Immunology, the First Affiliated Hospital of Anhui Medical University, Hefei, China. All cases were diagnosed by the skilled rheumatologist according to the modified 1984 New York Criteria [15]. Unrelated, ethnically matched healthy participants were selected as the control group. For each participant, 5ml of peripheral blood was obtained to extract genomic DNA for further sequencing and genotyping analysis. Disease activity of AS patients was measured by the Bath Ankylosing Spondylitis Disease Activity Index (BASDAI), scored from 0 to 10, with higher values indicating the more serious disease activity. Functional impairment was measured by the Bath Ankylosing Spondylitis Functional Index (BASFI), with a score from 0 to 10, where 0 was no function damage and 10 was the worse functional ability.

### Next generation sequencing

**Fig 1:**
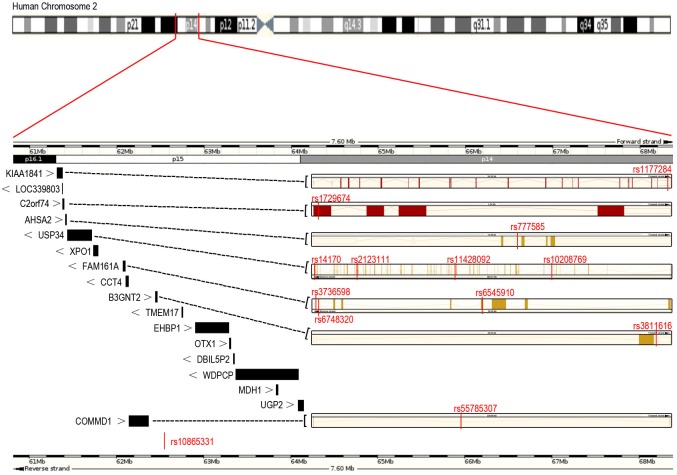
Schematic genomic structure on chromosome 2p15 region, and locations of the common single nucleotide polymorphisms (SNPs) identified in this study.

DNA was extracted from venous blood using a QIAGEN kit (QIAGEN, Hilden, Germany) according to the manufacturer’s instructions. In this study, we selected seventeen major genes containing *KIAA1841*, *LOC339803*, *C2orf74*, *AHSA2*, *USP34*, *XPO1*, *FAM161A*, *CCT4*, *COMMD1*, *B3GNT2*, *TMEM17*, *EHBP1*, *OTX1*, *DBIL5P2*, *WDPCP*, *MDH1*, and *UGP2* on chromosome 2p15 area(2,800,000bp, chr2:61,300,001–64,100,000, UCSC, GRCh37/hg19)(Fig 1). Fast Target^TM^ objective regional enrichment technology was used for the exon regional enrichment of candidate genes. And sequencing of objective regions were carried out using the Illumina MiSeqBenchtop Sequencer(Shanghai Genesky Bio-Tech Co, Ltd;www.geneskies.com). The quality of the output sequence data was assessed using FastQC (http://www.bioinformatics.babraham.ac.uk/projects/fastqc/) and sequencing adapters were trimmed using Trimmomatic. The 3'-end nucleotides with phred quality scores below 20 were trimmed using the fastx trimmer tool of FASTX toolkit (http://hannonlab.cshl.edu/fastx_toolkit). Trimmed pair-end reads were aligned by USEARCH and then compared with fragment reference sequences (hg19) using the Blat program. BWA (v0.7.5a) was used to map the reads, followed by SAM-to-BAM conversion, sorting, and removal of duplicates with SAMtools (v0.1.19). Combined SNP calling was performed on the resulting BAM files using GATK and Varscan programs. The Annovar program was used for SNP annotation. SNPs were filtered for association analysis using a Perl script to identify SNPs with greater than 5% allele frequency. Four different genetic models, which included additive model, dominant model, recessive model and allele model, were constructed for each of these SNPs. The principle of selecting targeted SNPs was based on the minimum *P* value among different genetic models. A two-sided *P*-value less than 0.2 was considered significant meaning and met the criteria for the validating stage.

### Follow-up genotyping by imLDR^TM^ technique

In this stage, we selected twelve promising SNPs identified in the first discovery phase (shown in S1 Table). Meanwhile, rs10865331 would also be further validated. Selected SNPs were genotyped using the improved multiplex ligase detection reaction (iMLDR) technique. This method was developed according to traditional ligase detection reaction by Genesky Biotechnologies Inc(Shanghai, China).In this study, a multiplex polymerase chain reaction (PCR)was designed to amplify the thirteen SNPs loci. And the primers were listed in S2 Table. Amplification products were used as the template for subsequent connections enzyme reaction after purified by nuclease and shrimp alkali enzyme (ExoI/SAP). In a connection the reaction, each site contains two 5' terminalallele specific probes and a 3' terminal specific probe of fluorescent tags. The ligation products were loaded in ABI3730XL, and the raw data were analyzed by GeneMapper4.1 (Applied Biosystems, USA).

### Statistical analysis

Statistical analysis was performed using SPSS version 16.0 (SPSS, Int., Chicago, IL). The differences in allele and genotype frequencies between patients and controls were assessed by the χ^2^ test. Odds ratios (ORs) with 95% confidence intervals (CIs) were calculated. Clinical phenotype (BASFI or BASDAI) comparisons among the three genotypic groups were conducted using Kruskal-Wallis *H* test. Logistic regression analysis was used to assess the association of each genotype with the risk for the disease. In addition, haplotype analysis was performed by software Haploview 4.2 [16]. Hardy–Weinberg Equilibrium (HWE) was evaluated in controls by χ^2^ analysis. A *P* value of less than 0.05 was considered statistically significant, and Bonferroni correction for multiple comparisons was applied when appropriate. *P* value for a truly significant result was calculated as 0.05/n.

## Results

### Identification of genetic variants in seventeen biological candidate genes

In the discovery phase, 100 AS patients (79 males and 21 females, mean age (SD) = 29.5 (9.7) years) and 100 healthy controls (80 males and 20 females, mean age (SD) = 29.91 (9.25) years) were enrolled and the two groups were comparable in the term of sex and age (*P* = 0.861, *P* = 0.760, respectively). In consideration of the exploratory nature of the first stage, a *P*-value less than 0.2 was considered statistically suggestive. Finally, 12 SNPs with *P*-value <0.2 (rs14170, rs11428092, rs10208769 and rs21231111 in *USP34*, rs6545910, rs6748320 and rs3736598 in *FAM161A*, rs777585 in *AHSA2*, rs3811616 in*B3GNT2*, rs1729674 in *C2orf74*, rs55785307 in *COMMD1*, and rs1177284 in *KIAA1841*) were identified and replicated in the follow-up study (S1 Table) (Fig 1). We did not find any novel SNPs.

### Associations between predisposed variants and AS

In the second stage, we replicated the 12 variants identified in the first stage and the positive locus rs10865331 identified by GWAS in 620 cases (515 males and 105 females) and 620 controls (512 males and 108 females). The mean age (SD) was 28.28 (8.92) years in patients and 27.83 (7.58) years in controls. Gender and age were comparable between the two groups (*P*> 0.05 for both).

All of the SNPs were in HWE in controls except rs10865331 (*P* = 0.047), rs2123111 (*P* = 0.028), rs6545910 (*P* = 0.042) and rs1177284 (*P* = 0.016). There were statistically significant differences in the genotype and allele frequencies at rs10865331 between AS patients and healthy controls (*P* = 0.006 and *P* = 0.001, respectively). There were no statistically significant differences in the genotype frequencies at other 12 SNPs loci but the allele frequencies showed a statistically significant association at rs14170 (*P* = 0.033), rs2123111 (*P* = 0.028) and rs1729674 (*P* = 0.037) (Table 1). However, only the difference of allele frequencies at rs10865331 remained statistically significant after Bonferroni correction. The results of subgroup analysis by gender showed that there were significant differences in the genotype frequencies at rs10865331, rs14170, rs2123111 (*P*<0.05 for all) and in allele frequencies at rs14170, rs11428092, rs2123111, rs1729674 and rs10865331between male AS patients and controls (*P*<0.05 for all) (S3 Table). Only the rs10865331 SNP showed significant difference in allele frequencies when comparing female AS patients to controls (*P* = 0.046) (S4 Table). However, these differences were no longer statistically significant after Bonferroni correction.

**Table 1:** The genotype and allele frequencies of identified SNPs in AS cases and healthy controls.

Gene	SNP	Genotype	Case	Control	*χ*^*2*^	*P*	Allele	Case	Control	OR (95%CI)	*χ*^*2*^	*P*
USP34	rs14170	A/A	268	309	5.346	0.069	A	810	861	1.202(1.015–1.423)	4.560	0.033
		A/G	274	243			G	424	375			
		G/G	75	66								
	rs11428092	-/-	282	261	3.816	0.148	-	835	795	0.857(0.725–1.013)	3.285	0.070
		-/A	271	273			A	397	441			
		A/A	63	84								
	rs10208769	A/A	279	310	3.026	0.220	A	823	861	1.147(0.968–1.358)	2.505	0.114
		A/T	265	241			T	411	375			
		T/T	73	67								
	rs2123111	G/G	280	321	5.335	0.069	G	823	875	1.210(1.021–1.435)	4.829	0.028
		G/A	263	233			A	411	361			
		A/A	74	64								
FAM161A	rs6545910	C/C	396	410	2.604	0.272	C	990	997	1.028(0.843–1.254)	0.075	0.784
		C/T	198	177			T	244	239			
		T/T	23	31								
	rs6748320	G/G	246	266	1.175	0.556	G	779	806	1.085(0.920–1.280)	0.946	0.331
		G/A	287	274			A	451	430			
		A/A	82	78								
	rs3736598	G/G	253	270	0.913	0.634	G	789	811	1.076(0.912–1.270)	0.761	0.383
		G/A	283	271			A	445	425			
		A/A	81	77								
AHSA2	rs777585	T/T	281	261	3.174	0.205	T	832	795	0.867(0.734–1.024)	2.834	0.092
		T/C	270	273			C	400	441			
		C/C	65	84								
B3GNT2	rs3811616	A/A	365	357	0.328	0.849	A	946	940	0.967(0.803–1.164)	0.127	0.722
		A/G	216	226			G	288	296			
		G/G	36	35								
C2orf74	rs1729674	T/T	266	303	4.490	0.106	T	803	853	1.195(1.011–1.414)	4.339	0.037
		T/G	271	247			G	431	383			
		G/G	80	68								
COMMD1	rs55785307	C/C	334	342	0.435	0.804	C	911	925	1.055(0.880–1.263)	0.332	0.564
		C/G	243	241			G	323	311			
		G/G	40	35								
KIAA1841	rs1177284	G/G	195	222	3.458	0.177	G	692	716	1.070(0.913–1.256)	0.701	0.403
		G/A	302	272			A	538	520			
		A/A	118	124								
__	rs10865331	G/G	178	216	10.084	0.006	G	628	710	1.303(1.111–1.526)	10.678	0.001^a^
		G/A	272	278			A	606	526			
		A/A	167	124								

SNP, Single nucleotide polymorphism

^a^ *P*-value remained statistically significant after Bonferroni correction.

Logistic regression analysis revealed that the AA genotype of rs10865331 in the gene desert area of 2p15 increased the risk of AS (OR (95% CI) = 1.634(1.204–2.218)) when compared with the GG genotype. In addition, rs14170 AG genotype and rs2123111 GA genotype was associated with a significantly increased risk of AS when compared with the AA genotype (OR (95% CI) = 1.300(1.025–1.649)) and GG genotype (OR (95% CI) = 1.294(1.020–1.642)) (Table 2).

**Table 2:** Association of thirteen SNPs with AS in a Chinese population.

Gene	SNP	Genotype	Case	Control	MAF		OR^a^(95%CI)	*P*^a^
					Case	Control		
USP34	rs14170	A/A	268	309	0.344	0.303		
		A/G	274	243			1.300(1.025–1.649)	0.031
		G/G	75	66			1.310(0.906–1.895)	0.151
	rs11428092	-/-	282	261	0.322	0.357		
		-/A	271	273			0.919(0.724–1.165)	0.919
		A/A	63	84			0.694(0.481–1.002)	0.052
	rs10208769	A/A	279	310	0.333	0.303		
		A/T	265	241			1.222(0.963–1.550)	0.099
		T/T	73	67			1.211(0.837–1.751)	0.310
	rs2123111	G/G	280	321	0.333	0.292		
		G/A	263	233			1.294(1.020–1.642)	0.034
		A/A	74	64			1.326(0.915–1.921)	0.136
FAM161A	rs6545910	C/C	396	410	0.198	0.193		
		C/T	198	177			1.158(0.906–1.480)	0.241
		T/T	23	31			0.768(0.440–1.340)	0.353
	rs6748320	G/G	246	266	0.367	0.348		
		G/A	287	274			1.133(0.891–1.439)	0.309
		A/A	82	78			1.137(0.797–1.622)	0.479
	rs3736598	G/G	253	270	0.361	0.344		
		G/A	283	271			1.114(0.877–1.415)	0.374
		A/A	81	77			1.123(0.786–1.603)	0.524
AHSA2	rs777585	T/T	281	261	0.325	0.357		
		T/C	270	273			0.919(0.724–1.166)	0.485
		C/C	65	84			0.719(0.499–1.035)	0.076
B3GNT2	rs3811616	A/A	365	357	0.233	0.239		
		A/G	216	226			0.935(0.738–1.185)	0.577
		G/G	36	35			1.006(0.618–1.638)	0.981
C2orf74	rs1729674	T/T	266	303	0.349	0.310		
		T/G	271	247			1.250(0.985–1.586)	0.067
		G/G	80	68			1.340(0.932–1.926)	0.114
COMMD1	rs55785307	C/C	334	342	0.262	0.252		
		C/G	243	241			1.032(0.818–1.304)	0.789
		G/G	40	35			1.170(0.726–1.887)	0.519
KIAA1841	rs1177284	G/G	195	222	0.437	0.421		
		G/A	302	272			1.264(0.982–1.627)	0.069
		A/A	118	124			1.083(0.789–1.488)	0.621
__	rs10865331	G/G	178	216	0.491	0.426		
		G/A	272	278			1.187(0.916–1.539)	0.195
		A/A	167	124			1.634(1.204–2.218)	0.002^b^

SNP, Single nucleotide polymorphism; MAF, minor allele frequency

^a^ ORs and *P* values were obtained from logistic regression analysis

^b^ *P*-value remained statistically signiciant after Bonferroni correction.

Furthermore, dominant, recessive and homozygous models were conducted between AS patients and healthy controls. Significant evidence was also detected under the dominant model for minor allele, and the minor allele carrier had an increased risk for AS compared with homozygous wild genotype carrier at rs10865331(OR(95% CI) = 1.325(1.042–1.685)), rs14170(OR(95% CI) = 1.302(1.041–1.629)), rs2123111(OR(95% CI) = 1.301(1.040–1.627)) and rs1729674(OR(95% CI) = 1.269(1.014–1.588)) (S5 Table). However, there was no significant difference after Bonferroni correction. Then, we repeated the three genetic models analysis after the gender stratification. For female AS cases and controls, no significant relationships were identified under dominant, recessive and homozygous models. However, there were significant differences among male AS cases and controls in the dominant model of rs14170 (OR(95% CI) = 1.379(1.078–1.763)), rs10208769 (OR(95% CI) = 1.304(1.020–1.668)), rs2123111 (OR(95% CI) = 1.368(1.070–1.749)), rs1729674 (OR(95% CI) = 1.357(1.061–1.736)) and rs10865331 (OR(95% CI) = 1.309(1.003–1.708)), in the recessive model of rs10865331 (OR(95% CI) = 1.405(1.050–1.879)). Also, we identified significant difference between male AS patients and controls in the homozygous model of rs11428092 (OR(95% CI) = 0.658(0.443–0.979)) and rs10865331 (OR(95% CI) = 1.564(1.115–2.193)) (See S6 Table). Similarly, the associations were not statistically significant after Bonferroni correction.

Linkage disequilibrium (LD) analysis showed a strong LD among rs1729674, rs14170, rs2123111and rs10208769 (D’> 0.90 and r^2^> 0.80) (Table 3). These four SNPs were used to construct two haplotypes: TAGA and GGAT. The haplotype TAGA was associated with a decreased risk for AS (OR(95% CI) = 0.832(0.705–0.982)) (Table 4). Haplotypes with frequency less than 0.03 were not considered in analysis.

**Table 3:** Pairwise linkage disequilibrium (LD) results among SNPs rs1729674, rs14170, rs2123111 and rs10208769.

SNPs	rs1729674	rs14170	rs2123111	rs10208769
rs1729674		0.968	0.982	0.944
rs14170	0.911		0.984	0.952
rs2123111	0.892	0.922		0.973
rs10208769	0.846	0.885	0.922	

SNP, Single nucleotide polymorphism

D’ and r^2^ values are shown above and below the diagonal, respectively.

**Table 4:** Haplotype frequencies in AS and controls.

Haplotypes	Cases	Controls	*χ*^*2*^	*P* value	OR (95% CI)
TAGA	0.631	0.673	4.741	0.029	0.832(0.705–0.982)
GGAT	0.317	0.290	2.105	0.147	1.136(0.957–1.348)

OR, Odds ratio; CI, Confidence interval

The order of the polymorphisms is according to the positions on the chromosome: rs1729674, rs14170, rs2123111 and rs10208769.

We also investigated the association between the different genotypes and clinical phenotypes including the BASDAI and BASFI. As shown in S7 Table, we found no relationship between the 13 SNPs and the susceptibility and severity of AS.

## Discussion

Genetic factors play a critical role in the pathogenesis of AS. Previous GWAS and large-scale case-control studies have demonstrated that, in addition to the major histocompatibility complex (MHC) HLA-B27, multiple non-MHC susceptibility genes or area, such as *ERAP1*, *IL23R* and some susceptible gene desert area, might play a paramount role in disease susceptibility [8, 17–20].

Our group has been studying genetic factors associated with AS susceptibility for several years [21–23]. In the present study, we carried out a two-step study to investigate whether 2p15 area contributed to the development of AS. In the first discovery stage, we selected 17 major genes on chromosome 2p15 area as candidate genes, with 12 SNPs being identified to be the potentially functional genetic variants for AS. Importantly, the 12 SNPs have not been reported in the previous studies with several thousand patients and controls. In the second validated stage, we for the first time examined the correlation between 13 SNPs (12 identified SNPs and rs10865331) and the susceptibility to AS in a Chinese population. Among the 12 SNPs identified in the first stage, three (rs14170 and rs2123111 in *USP34*, rs1729674 in *C2orf74*) were likely to be associated with AS susceptibility though the differences were no longer statistically significant after the Bonferroni correction. Moreover, stratification analysis by gender further indicated that another two SNPs (rs11428092 and rs10208769 in *USP34*) might confer susceptibilities to AS, providing further evidence of the association between *USP34* and AS susceptibility. Furthermore, rs1729674, rs14170, rs2123111 and rs10208769 were in high LD and included in haplotype analysis. We found a novel haplotype (TAGA) which might play a protective role in AS susceptibility. We also demonstrated a strong relationship between rs10865331 and AS susceptibility, which has been widely confirmed in different ethnic populations [9, 12, 13]. Notably, there was no LD between rs10865331 and rs1729674, rs14170, rs2123111. Thus, we inferred that the possible association between the three SNPs (rs14170 and rs2123111 in USP34, rs1729674 in C2orf74) and AS susceptibility was not affected by rs10865331.

2p15 region contains genes desert area and a large number of genes. The association between 2p15 and AS susceptibility has been widely reported [8, 10]; however, the positive locus rs10865331 is located in gene desert area, which refers to the intergenic regions on chromosome approximately accounting for 25% of the genome. These segments are not responsible for any protein coding, and their functional significance remains elusive [24]. In this study, we provided first evidence that rs14170 and rs2123111 in *USP34*, and rs1729674 in *C2orf74* might be associated with AS susceptibility. In addition, evidence from stratification analysis supported the conclusion that *USP34* might play a role in AS. Although the significance disappeared after Bonferroni correction, as a strict multiple correction methods, Bonferroni correction may obtain far conservative conclusion especially in the presence of a large number of SNPs [25]. Indeed, none of the identified SNPs survived from Bonferroni correction except the rs10865331, with only suggestive evidence. It might be explained by the small sample size given relatively small magnitude for the associations of these SNPs with AS. Although the evidence was suggestive, the current study was valuable as it for the first time performed sequencing on 2p15 using NGS with all variants. Nonetheless, these associations should be further verified in future studies with much larger samples.

Suspicious positive SNPs including rs14170 and rs2123111 were located in *USP34* gene and had a strong LD (D’ = 0.984, r^2^ = 0.922). *USP34*, as a protein coding gene, encodes a kind of deubiquitinating enzyme, which belongs to ubiquitin-specific protease family. It has been reported that somatic variations of *USP34* are related to ovary tumor [26]. Moreover, *USP34* positively regulates Wnt signaling pathway [27] and plays a role in DNA damage response control [28]. However, biological explanations for the associations of *USP34* variants with AS have not yet been forthcoming. Poalas et al. reported that *USP34* played a role in NFκB signal regulation in T lymphocytes [29]. Therefore, it is possible that *USP34* may indirectly participate in the pathophysiology of AS by adjusting NFκB signal pathways, which have influences on the pathogenesis of AS [30]. However, further studies are still needed to evaluate the function of *USP34* in the process of AS.

Although it has been suggested that 2p15 plays an important role in AS, this is the first study to provide evidence of an association between the gene polymorphisms besides 2p15 genes desert area and AS susceptibility in a Chinese population. Our previous study has verified the IL-12B Polymorphisms susceptibility with AS in mainland Han population [31]. However, the previous study just analyzed some SNPs of a certain gene and focused on IL-12B gene, which is located in non-HLA region of chromosome 5q31–33. In contrast, the current study mainly paid attention to the gene desert area of chromosome 2p15, and found two potential susceptibility genes and corresponding genetic susceptibility loci, which would make a big progress in the association study of AS. However, some limitations of the present study should be mentioned. First, the sample size of the second validated stage was relatively small. In addition, the recruited participants were all Han Chinese and the results might not represent other ethnic populations.

In summary, our two-stage genetic association study for the first time provides evidence that the rs14170 and rs2123111 in*USP34* and rs1729674 in *C2orf74* may be associated with AS susceptibility in a Han Chinese population. *USP34* and *C2orf74* may be AS novel susceptibility gene in 2p15 region. Further studies with larger sample size are required to confirm these associations in other ethnics.

## Supporting information

S1 Table Genetic variations identified in the candidate genes on chromosome 2p15 through next generation sequencing. (DOCX)

S2 Table The information of PCR primer for SNPs genotyping. (DOCX)

S3 Table The distribution of genotype and allele of identified SNPs in male AS cases and male healthy controls. (DOCX)

S4 Table The distribution of genotype and allele of identified SNPs in female AS cases and female healthy controls. (DOCX)

S5 Table The genotype frequencies of identified SNPs in dominant genetic models in AS cases and controls. (DOCX)

S6 Table Results of the analyses in different genetic models in males. (DOCX)

S7 Table BASDAI and BASFI scores in AS patients with different genotypes. (DOCX)
