# Supplementary material for: Pathogenic variants screening in seventeen candidate genes on 2p15 for association with ankylosing spondylitis in a Han Chinese population
Source: PLoS One. 2017 May 11;12(5):e0177080. doi: 10.1371/journal.pone.0177080 (PMC5426703; doi:10.1371/journal.pone.0177080)
Supplement: S1 Table — (DOCX) [file pone.0177080.s001.docx]

**S1Table. Genetic variations identified in the candidate genes on chromosome 2p15 through next generation sequencing**

| Gene | SNP ID | Position | Alleles | Gene Region | Sample MAF | Population MAF  (from 1000 genomes) | *P*^a^ |
| --- | --- | --- | --- | --- | --- | --- | --- |
| USP34 | rs14170 | 61415492 | A/G | exonic | 0.32 | 0.46 | 0.147 |
|  | rs11428092 | 61528299 | -/A | splicing | 0.29 | 0.30 | 0.010 |
|  | rs10208769 | 61605614 | A/T | intronic | 0.32 | 0.50 | 0.086 |
|  | rs2123111 | 61450454 | A/G | intronic | 0.31 | 0.46 | 0.087 |
| FAM161A | rs6545910 | 62065759 | C/T | exonic | 0.22 | 0.12 | 0.114 |
|  | rs6748320 | 62053290 | A/G | 3'UTR | 0.35 | 0.45 | 0.142 |
|  | rs3736598 | 62052380 | A/G | 3'UTR | 0.34 | 0.46 | 0.128 |
| AHSA2 | rs777585 | 61412559 | C/T | intronic | 0.32 | 0.33 | 0.155 |
| B3GNT2 | rs3811616 | 62450631 | A/G | 3'UTR | 0.20 | 0.19 | 0.170 |
| C2orf74 | rs1729674 | 61389737 | G/T | exonic; splicing | 0.34 | 0.43 | 0.147 |
| COMMD1 | rs55785307 | 62228180 | C/G | intronic | 0.31 | 0.21 | 0.193 |
| KIAA1841 | rs1177284 | 61349446 | A/G | 3'UTR | 0.44 | 0.29 | 0.071 |

SNP, Single nucleotide polymorphism

^a^ The minimum *P*-value was obtained from different genetic association models (including additive model, dominant model, recessive model and allele model).
