# Supplementary material for: Pathogenic variants screening in seventeen candidate genes on 2p15 for association with ankylosing spondylitis in a Han Chinese population
Source: PLoS One. 2017 May 11;12(5):e0177080. doi: 10.1371/journal.pone.0177080 (PMC5426703; doi:10.1371/journal.pone.0177080)
Supplement: S2 Table — (DOCX) [file pone.0177080.s002.docx]

**S2 Table. The information of PCR primer for SNPs genotyping**

| SNP ID | Chromosome position | PCR primer |
| --- | --- | --- |
| rs14170 | 61415492 | rs14170F:  AGCTTGGCCATCACAGCTTCTC |
|  |  | rs14170R:  ACCCCAACGAGCACTTCTGACA |
| rs11428092 | 61528299 | rs11428092F:  ACCTTCCCCTTTCCGCTCTCTC |
|  |  | rs11428092R:  CATGGGACCTGTCAGGATGATTTC |
| rs10208769 | 61605614 | rs10208769F:  TTGCACACATCATTGAAGGTATGG |
|  |  | rs10208769R:  CTGCGCCTGGCCATTATTTAGT |
| rs2123111 | 61450454 | rs2123111F:  AATGCTGCCATGTCAGTCTGTGA |
|  |  | rs2123111R:  GGAATGTTGAAAAGTTGCCCAAAA |
| rs6545910 | 62065759 | rs6545910F:  ACCAGTGGTCTGGAGATTCAAGATTT |
|  |  | rs6545910R:  GTTGGAAGAAGAGAGAAATCGGATCC |
| rs6748320 | 62053290 | rs6748320F:  CTCAGAGCAGCAAAACAAGTTAGAGGATTT |
|  |  | rs6748320R:  ATGTGCAGGGCTGTTGATCAAAGTC |
| rs3736598 | 62052380 | rs3736598F:  AAACATTTGCCCCTGATGGAGA |
|  |  | rs3736598R:  TCACTGCTAGACCCAGTTGTGTTCA |
| rs777585 | 61412559 | rs777585F:  CTTTCACCTGCTCACGGCTGTA |
|  |  | rs777585R:  GGGAAGAGGTTCCAGGGAATGAT |
| rs3811616 | 62450631 | rs3811616F:  GAAACTCGGCCTCGTTCCAGAG |
|  |  | rs3811616R:  AGAGGCCCTCAAATGGGTATCA |
| rs1729674 | 61389737 | rs1729674F:  TTGCCTCATTTGCATCCTCCTC |
|  |  | rs1729674R:  TGGCCCTGATTTTAAGGTGTGGT |
| rs55785307 | 62228180 | rs55785307F:  GGGCCTGAGCTGGAGAGTTGAT |
|  |  | rs55785307R:  TGCCAGGACCCTGAGGCTAGTT |
| rs1177284 | 61349446 | rs1177284F:  GGTCAAGGGCGTCCTGCATAA |
|  |  | rs1177284R:  GGCTACCTGACAGTTTAGGCCTTTC |
| rs10865331 | 62551472 | rs10865331F:  GCCCTGGCTGGTGATTTTCTTT |
|  |  | rs10865331R:  TTGAGGCAATGGCCACTTTACG |

SNP, Single nucleotide polymorphism; PCR: polymerase chain reaction; F: forward; R: reverse
