# Supplementary material for: Pathogenic variants screening in seventeen candidate genes on 2p15 for association with ankylosing spondylitis in a Han Chinese population
Source: PLoS One. 2017 May 11;12(5):e0177080. doi: 10.1371/journal.pone.0177080 (PMC5426703; doi:10.1371/journal.pone.0177080)
Supplement: S3 Table — (DOCX) [file pone.0177080.s003.docx]

**S3 Table. The distribution of genotype and allele of identified SNPs in male AS cases and male healthy controls**

| Gene | SNPs |  | Genotype | | *χ^2^* | *P* |  | Allele | | *OR* (95%*CI*) | *χ^2^* | *P* |
| --- | --- | --- | --- | --- | --- | --- | --- | --- | --- | --- | --- | --- |
|  |  |  | Case | Control |  |  |  | Case | Control |  |  |  |
| USP34 | rs14170 | A/A | 223 | 262 | 6.658 | 0.036 | A | 677 | 718 | 1.233 (1.023, 1.485) | 4.851 | 0.028 |
|  |  | A/G | 231 | 194 |  |  | G | 351 | 302 |  |  |  |
|  |  | G/G | 60 | 54 |  |  |  |  |  |  |  |  |
|  | rs11428092 | -/- | 238 | 208 | 4.721 | 0.094 | - | 696 | 645 | 0.816 (0.679, 0.979) | 4.794 | 0.029 |
|  |  | -/A | 220 | 229 |  |  | A | 330 | 375 |  |  |  |
|  |  | A/A | 55 | 73 |  |  |  |  |  |  |  |  |
|  | rs10208769 | A/A | 233 | 265 | 4.596 | 0.100 | A | 687 | 720 | 1.191 (0.988, 1.436) | 3.365 | 0.067 |
|  |  | A/T | 221 | 190 |  |  | T | 341 | 300 |  |  |  |
|  |  | T/T | 60 | 55 |  |  |  |  |  |  |  |  |
|  | rs2123111 | G/G | 234 | 272 | 6.244 | 0.044 | G | 687 | 730 | 1.249 (1.035, 1.508) | 5.396 | 0.020 |
|  |  | G/A | 219 | 186 |  |  | A | 341 | 290 |  |  |  |
|  |  | A/A | 61 | 52 |  |  |  |  |  |  |  |  |
| FAM161A | rs6545910 | C/C | 335 | 343 | 1.577 | 0.454 | C | 828 | 827 | 1.035 (0.831, 1.290) | 0.094 | 0.759 |
|  |  | C/T | 158 | 141 |  |  | T | 200 | 193 |  |  |  |
|  |  | T/T | 21 | 26 |  |  |  |  |  |  |  |  |
|  | rs6748320 | G/G | 202 | 216 | 1.079 | 0.583 | G | 643 | 663 | 1.100 (0.919, 1.318) | 1.079 | 0.299 |
|  |  | G/A | 239 | 231 |  |  | A | 381 | 357 |  |  |  |
|  |  | A/A | 71 | 63 |  |  |  |  |  |  |  |  |
|  | rs3736598 | G/G | 208 | 220 | 0.864 | 0.649 | G | 652 | 667 | 1.090 (0.909, 1.306) | 0.865 | 0.352 |
|  |  | G/A | 236 | 227 |  |  | A | 376 | 353 |  |  |  |
|  |  | A/A | 70 | 63 |  |  |  |  |  |  |  |  |
| AHSA2 | rs777585 | T/T | 235 | 208 | 3.535 | 0.171 | T | 690 | 645 | 0.838 (0.698, 1.005) | 3.639 | 0.056 |
|  |  | T/C | 220 | 229 |  |  | C | 336 | 375 |  |  |  |
|  |  | C/C | 58 | 73 |  |  |  |  |  |  |  |  |
| B3GNT2 | rs3811616 | A/A | 307 | 302 | 0.228 | 0.892 | A | 791 | 785 | 1.001 (0.815, 1.229) | 0.000 | 0.993 |
|  |  | A/G | 177 | 181 |  |  | G | 237 | 235 |  |  |  |
|  |  | G/G | 30 | 27 |  |  |  |  |  |  |  |  |
| C2orf74 | rs1729674 | T/T | 222 | 259 | 5.927 | 0.052 | T | 671 | 713 | 1.236 (1.027, 1.487) | 5.008 | 0.025 |
|  |  | T/G | 227 | 195 |  |  | G | 357 | 307 |  |  |  |
|  |  | G/G | 65 | 56 |  |  |  |  |  |  |  |  |
| COMMD1 | rs55785307 | C/C | 279 | 280 | 0.071 | 0.965 | C | 760 | 759 | 1.025 (0.841, 1.250) | 0.062 | 0.803 |
|  |  | C/G | 202 | 199 |  |  | G | 268 | 261 |  |  |  |
|  |  | G/G | 33 | 31 |  |  |  |  |  |  |  |  |
| KIAA1841 | rs1177284 | G/G | 162 | 191 | 4.428 | 0.109 | G | 574 | 601 | 1.125 (0.944, 1.340) | 1.718 | 0.190 |
|  |  | G/A | 250 | 219 |  |  | A | 450 | 419 |  |  |  |
|  |  | A/A | 100 | 100 |  |  |  |  |  |  |  |  |
| __ | rs10865331 | G/G | 143 | 171 | 6.748 | 0.034 | G | 521 | 577 | 1.267 (1.065, 1.508) | 7.137 | 0.008 |
|  |  | G/A | 235 | 235 |  |  | A | 507 | 443 |  |  |  |
|  |  | A/A | 136 | 104 |  |  |  |  |  |  |  |  |

SNP, Single nucleotide polymorphism
