# Supplementary material for: Pathogenic variants screening in seventeen candidate genes on 2p15 for association with ankylosing spondylitis in a Han Chinese population
Source: PLoS One. 2017 May 11;12(5):e0177080. doi: 10.1371/journal.pone.0177080 (PMC5426703; doi:10.1371/journal.pone.0177080)
Supplement: S4 Table — (DOCX) [file pone.0177080.s004.docx]

**S4 Table. The distribution of genotype and allele of identified SNPs in female AS cases and female healthy controls**

| Gene | SNPs |  | Genotype | | *χ^2^* | *P* |  | Allele | | *OR* (95%*CI*) | *χ^2^* | *P* |
| --- | --- | --- | --- | --- | --- | --- | --- | --- | --- | --- | --- | --- |
|  |  |  | Case | Control |  |  |  | Case | Control |  |  |  |
| USP34 | rs14170 | A/A | 45 | 47 | 0.650 | 0.723 | A | 133 | 143 | 1.075 (0.720, 1.606) | 0.125 | 0.723 |
|  |  | A/G | 43 | 49 |  |  | G | 73 | 73 |  |  |  |
|  |  | G/G | 15 | 12 |  |  |  |  |  |  |  |  |
|  | rs11428092 | -/- | 44 | 53 | 1.707 | 0.426 | - | 139 | 150 | 1.095 (0.726, 1.652) | 0.189 | 0.663 |
|  |  | -/A | 51 | 44 |  |  | A | 67 | 66 |  |  |  |
|  |  | A/A | 8 | 11 |  |  |  |  |  |  |  |  |
|  | rs10208769 | A/A | 46 | 45 | 0.449 | 0.799 | A | 136 | 141 | 0.968 (0.647, 1.446) | 0.026 | 0.873 |
|  |  | A/T | 44 | 51 |  |  | T | 70 | 75 |  |  |  |
|  |  | T/T | 13 | 12 |  |  |  |  |  |  |  |  |
|  | rs2123111 | G/G | 46 | 49 | 0.115 | 0.944 | G | 136 | 145 | 1.051 (0.701, 1.575) | 0.058 | 0.809 |
|  |  | G/A | 44 | 47 |  |  | A | 70 | 71 |  |  |  |
|  |  | A/A | 13 | 12 |  |  |  |  |  |  |  |  |
| FAM161A | rs6545910 | C/C | 61 | 67 | 1.660 | 0.436 | C | 162 | 170 | 1.004 (0.630, 1.600) | 0.000 | 0.987 |
|  |  | C/T | 40 | 36 |  |  | T | 44 | 46 |  |  |  |
|  |  | T/T | 2 | 5 |  |  |  |  |  |  |  |  |
|  | rs6748320 | G/G | 44 | 50 | 1.155 | 0.561 | G | 136 | 143 | 1.008 (0.674, 1.509) | 0.002 | 0.968 |
|  |  | G/A | 48 | 43 |  |  | A | 70 | 73 |  |  |  |
|  |  | A/A | 11 | 15 |  |  |  |  |  |  |  |  |
|  | rs3736598 | G/G | 45 | 50 | 0.604 | 0.739 | G | 137 | 144 | 1.007 (0.672, 1.510) | 0.001 | 0.972 |
|  |  | G/A | 47 | 44 |  |  | A | 69 | 72 |  |  |  |
|  |  | A/A | 11 | 14 |  |  |  |  |  |  |  |  |
| AHSA2 | rs777585 | T/T | 46 | 53 | 1.649 | 0.438 | T | 142 | 150 | 1.024 (0.677, 1.549) | 0.013 | 0.909 |
|  |  | T/C | 50 | 44 |  |  | C | 64 | 66 |  |  |  |
|  |  | C/C | 7 | 11 |  |  |  |  |  |  |  |  |
| B3GNT2 | rs3811616 | A/A | 58 | 55 | 0.676 | 0.713 | A | 155 | 155 | 0.836 (0.542, 1.290) | 0.656 | 0.418 |
|  |  | A/G | 39 | 45 |  |  | G | 51 | 61 |  |  |  |
|  |  | G/G | 6 | 8 |  |  |  |  |  |  |  |  |
| C2orf74 | rs1729674 | T/T | 44 | 44 | 0.882 | 0.643 | T | 132 | 140 | 1.033 (0.693, 1.539) | 0.025 | 0.874 |
|  |  | T/G | 44 | 52 |  |  | G | 74 | 76 |  |  |  |
|  |  | G/G | 15 | 12 |  |  |  |  |  |  |  |  |
| COMMD1 | rs55785307 | C/C | 55 | 62 | 1.131 | 0.568 | C | 151 | 166 | 1.209 (0.777, 1.881) | 0.711 | 0.399 |
|  |  | C/G | 41 | 42 |  |  | G | 55 | 50 |  |  |  |
|  |  | G/G | 7 | 4 |  |  |  |  |  |  |  |  |
| KIAA1841 | rs1177284 | G/G | 33 | 31 | 0.811 | 0.667 | G | 118 | 115 | 0.849 (0.578, 1.247) | 0.696 | 0.404 |
|  |  | G/A | 52 | 53 |  |  | A | 88 | 101 |  |  |  |
|  |  | A/A | 18 | 24 |  |  |  |  |  |  |  |  |
| __ | rs10865331 | G/G | 35 | 45 | 3.956 | 0.138 | G | 107 | 133 | 1.483 (1.007, 2.184) | 3.989 | 0.046 |
|  |  | G/A | 37 | 43 |  |  | A | 99 | 83 |  |  |  |
|  |  | A/A | 31 | 20 |  |  |  |  |  |  |  |  |

SNP, Single nucleotide polymorphism
