# Supplementary material for: Pathogenic variants screening in seventeen candidate genes on 2p15 for association with ankylosing spondylitis in a Han Chinese population
Source: PLoS One. 2017 May 11;12(5):e0177080. doi: 10.1371/journal.pone.0177080 (PMC5426703; doi:10.1371/journal.pone.0177080)
Supplement: S5 Table — (DOCX) [file pone.0177080.s005.docx]

**S5 Table. The genotype frequencies of** **identified SNPs in dominant genetic models in AS cases and controls**

| SNP | Allele | The dominant model | | | | |  |
| --- | --- | --- | --- | --- | --- | --- | --- |
|  |  | Genotype | Case | Control | OR(95%CI) | *χ^2^* | *P* |
| rs14170 | A/G | A/A | 268 | 309 | 1.302(1.041-1.629) | 5.344 | 0.021 |
|  |  | A/G+G/G | 349 | 309 |  |  |  |
| rs11428092 | -/A | -/- | 282 | 261 | 0.866(0.691-1.084) | 1.574 | 0.210 |
|  |  | -/A+A/A | 334 | 357 |  |  |  |
| rs10208769 | A/T | A/A | 279 | 310 | 1.219(0.975-1.525) | 3.024 | 0.082 |
|  |  | A/T+T/T | 338 | 308 |  |  |  |
| rs2123111 | G/A | G/G | 280 | 321 | 1.301(1.040-1.627) | 5.320 | 0.021 |
|  |  | G/A+A/A | 337 | 297 |  |  |  |
| rs6545910 | C/T | C/C | 396 | 410 | 1.100(0.870-1.391) | 0.636 | 0.425 |
|  |  | C/T+T/T | 221 | 208 |  |  |  |
| rs6748320 | G/A | G/G | 246 | 266 | 1.134(0.904-1.422) | 1.175 | 0.278 |
|  |  | G/A+A/A | 369 | 352 |  |  |  |
| rs3736598 | G/A | G/G | 253 | 270 | 1.116(0.891-1.399) | 0.911 | 0.340 |
|  |  | G/A+A/A | 364 | 348 |  |  |  |
| rs777585 | T/C | T/T | 281 | 261 | 0.872(0.696-1.091) | 1.434 | 0.231 |
|  |  | T/C+C/C | 335 | 357 |  |  |  |
| rs3811616 | A/G | A/A | 365 | 357 | 0.944(0.753-1.184) | 0.246 | 0.620 |
|  |  | A/G+G/G | 252 | 261 |  |  |  |
| rs1729674 | T/G | T/T | 266 | 303 | 1.269(1.014-1.588) | 4.351 | 0.037 |
|  |  | T/G+G/G | 351 | 315 |  |  |  |
| rs55785307 | C/G | C/C | 334 | 342 | 1.050(0.839-1.314) | 0.182 | 0.670 |
|  |  | C/G+G/G | 283 | 276 |  |  |  |
| rs1177284 | G/A | G/G | 195 | 222 | 1.207(0.953-1.529) | 2.447 | 0.118 |
|  |  | G/A+A/A | 420 | 396 |  |  |  |
| rs10865331 | G/A | G/G | 178 | 216 | 1.325(1.042-1.685) | 5.292 | 0.021 |
|  |  | G/A+A/A | 439 | 402 |  |  |  |

SNP, Single nucleotide polymorphism
