# Supplementary material for: Pathogenic variants screening in seventeen candidate genes on 2p15 for association with ankylosing spondylitis in a Han Chinese population
Source: PLoS One. 2017 May 11;12(5):e0177080. doi: 10.1371/journal.pone.0177080 (PMC5426703; doi:10.1371/journal.pone.0177080)
Supplement: S6 Table — (DOCX) [file pone.0177080.s006.docx]

**S6 Table. Results of the analyses in different genetic models in males**

| SNPs | Allele | Model | Genotype | AS | | Control | | | *OR* (95%*CI*) | *χ^2^* | | *P* |
| --- | --- | --- | --- | --- | --- | --- | --- | --- | --- | --- | --- | --- |
| rs14170 | A/G | Dominant model | A/A vs. A/G+G/G | 223 | 291 | | 262 | 248 | 1.379 (1.078, 1.763) | | 6.551 | 0.010 |
|  |  | recessive model | A/A+A/G vs. G/G | 454 | 60 | | 456 | 54 | 1.116 (0.756, 1.648) | | 0.305 | 0.581 |
|  |  | Homozygous model | A/A vs. G/G | 223 | 60 | | 262 | 54 | 1.305 (0.867, 1.965) | | 1.639 | 0.200 |
| rs11428092 | -/A | Dominant model | -/- vs. -/A+A/A | 238 | 275 | | 208 | 302 | 0.796 (0.621, 1.019) | | 3.273 | 0.070 |
|  |  | recessive model | -/-+-/A vs. A/A | 458 | 55 | | 437 | 73 | 0.719 (0.495, 1.045) | | 3.015 | 0.082 |
|  |  | Homozygous model | -/- vs. A/A | 238 | 55 | | 208 | 73 | 0.658 (0.443, 0.979) | | 4.300 | 0.038 |
| rs10208769 | A/T | Dominant model | A/A vs. A/T+T/T | 233 | 281 | | 265 | 245 | 1.304 (1.020, 1.668) | | 4.505 | 0.034 |
|  |  | recessive model | A/A+A/T vs. T/T | 454 | 60 | | 455 | 55 | 1.093 (0.741, 1.612) | | 0.203 | 0.652 |
|  |  | Homozygous model | A/A vs. T/T | 233 | 60 | | 265 | 55 | 1.241 (0.827, 1.862) | | 1.086 | 0.297 |
| rs2123111 | G/A | Dominant model | G/G vs. G/A+A/A | 234 | 280 | | 272 | 238 | 1.368 (1.070, 1.749) | | 6.244 | 0.012 |
|  |  | recessive model | G/G+G/A vs. A/A | 453 | 61 | | 458 | 52 | 1.186 (0.801, 1.755) | | 0.729 | 0.393 |
|  |  | Homozygous model | G/G vs. A/A | 234 | 61 | | 272 | 52 | 1.364 (0.906, 2.053) | | 2.217 | 0.137 |
| rs6545910 | C/T | Dominant model | C/C vs. C/T+T/T | 335 | 179 | | 343 | 167 | 1.097 (0.847, 1.422) | | 0.495 | 0.482 |
|  |  | recessive model | C/C+C/T vs. T/T | 493 | 21 | | 484 | 26 | 0.793 (0.440, 1.428) | | 0.599 | 0.439 |
|  |  | Homozygous model | C/C vs. T/T | 335 | 21 | | 343 | 26 | 0.827 (0.456, 1.498) | | 0.393 | 0.531 |
| rs6748320 | G/A | Dominant model | G/G vs. G/A+A/A | 202 | 310 | | 216 | 294 | 1.128 (0.879, 1.447) | | 0.889 | 0.346 |
|  |  | recessive model | G/G+G/A vs. A/A | 441 | 71 | | 447 | 63 | 1.142 (0.794, 1.644) | | 0.514 | 0.473 |
|  |  | Homozygous model | G/G vs. A/A | 202 | 71 | | 216 | 63 | 1.205 (0.816, 1.779) | | 0.881 | 0.348 |
| rs3736598 | G/A | Dominant model | G/G vs. G/A+A/A | 208 | 306 | | 220 | 290 | 1.116 (0.871, 1.431) | | 0.750 | 0.386 |
|  |  | recessive model | G/G+G/A vs. A/A | 444 | 70 | | 447 | 63 | 1.119 (0.777, 1.611) | | 0.363 | 0.547 |
|  |  | Homozygous model | G/G vs. A/A | 208 | 70 | | 220 | 63 | 1.175 (0.796, 1.735) | | 0.660 | 0.416 |
| rs777585 | T/C | Dominant model | T/T vs. T/C+C/C | 235 | 278 | | 208 | 302 | 0.815 (0.636, 1.044) | | 2.630 | 0.105 |
|  |  | recessive model | T/T+T/C vs. C/C | 458 | 58 | | 437 | 73 | 0.763 (0.528, 1.104) | | 2.072 | 0.150 |
|  |  | Homozygous model | T/T vs. C/C | 235 | 58 | | 208 | 73 | 0.703 (0.475, 1.041) | | 3.114 | 0.078 |
| rs3811616 | A/G | Dominant model | A/A vs. A/G+G/G | 307 | 207 | | 302 | 208 | 0.979 (0.763, 1.256) | | 0.028 | 0.867 |
|  |  | recessive model | A/A+A/G vs. G/G | 484 | 30 | | 483 | 27 | 1.109 (0.649, 1.893) | | 0.143 | 0.705 |
|  |  | Homozygous model | A/A vs. G/G | 307 | 30 | | 302 | 27 | 1.093 (0.635, 1.882) | | 0.103 | 0.748 |
| rs1729674 | T/G | Dominant model | T/T vs. T/G+G/G | 222 | 292 | | 259 | 251 | 1.357 (1.061, 1.736) | | 5.926 | 0.015 |
|  |  | recessive model | T/T+T/G vs. G/G | 449 | 65 | | 454 | 56 | 1.174 (0.802, 1.717) | | 0.681 | 0.409 |
|  |  | Homozygous model | T/T vs. G/G | 222 | 65 | | 259 | 56 | 1.354 (0.908, 2.020) | | 2.218 | 0.136 |
| rs55785307 | C/G | Dominant model | C/C vs. C/G+G/G | 279 | 235 | | 280 | 230 | 1.025 (0.802, 1.311) | | 0.040 | 0.842 |
|  |  | recessive model | C/C+C/G vs. G/G | 481 | 33 | | 479 | 31 | 1.060 (0.639, 1.759) | | 0.051 | 0.821 |
|  |  | Homozygous model | C/C vs. G/G | 279 | 33 | | 280 | 31 | 1.068 (0.637, 1.792) | | 0.063 | 0.802 |
| rs1177284 | G/A | Dominant model | G/G vs. G/A+A/A | 162 | 350 | | 191 | 319 | 1.294 (0.999, 1.675) | | 3.815 | 0.051 |
|  |  | recessive model | G/G+G/A vs. A/A | 412 | 100 | | 410 | 100 | 0.995 (0.731, 1.356) | | 0.001 | 0.975 |
|  |  | Homozygous model | G/G vs. A/A | 162 | 100 | | 191 | 100 | 1.179 (0.833, 1.669) | | 0.864 | 0.353 |
| rs10865331 | G/A | Dominant model | G/G vs. G/A+A/A | 143 | 371 | | 171 | 339 | 1.309 (1.003, 1.708) | | 3.924 | 0.048 |
|  |  | recessive model | G/G+G/A vs. A/A | 378 | 136 | | 406 | 104 | 1.405 (1.050, 1.879) | | 5.251 | 0.022 |
|  |  | Homozygous model | G/G vs. A/A | 143 | 136 | | 171 | 104 | 1.564 (1.115, 2.193) | | 6.735 | 0.009 |

SNP, Single nucleotide polymorphism
