# Supplementary material for: Pathogenic variants screening in seventeen candidate genes on 2p15 for association with ankylosing spondylitis in a Han Chinese population
Source: PLoS One. 2017 May 11;12(5):e0177080. doi: 10.1371/journal.pone.0177080 (PMC5426703; doi:10.1371/journal.pone.0177080)
Supplement: S7 Table — (DOCX) [file pone.0177080.s007.docx]

**S7 Table. BASDAI and BASFI scores in AS patients with different genotypes**

| SNPs | Genotype | BASDAI (Median (P_25_- P_75_)) | BASFI (Median (P_25_-P_75_)) |
| --- | --- | --- | --- |
| rs14170 | A/A | 1.800 (0.300-3.630) | 0.700 (0.000-2.100) |
|  | A/G | 1.600 (0.040-2.900) | 1.000 (0.000-2.765) |
|  | G/G | 2.115 (0.510-3.262) | 0.900 (0.200-2.100) |
|  | *P* | 0.443 | 0.081 |
| rs11428092 | -/- | 2.000 (0.200-3.400) | 0.900 (0.000-2.425) |
|  | -/A | 1.700 (0.400-3.100) | 0.900 (0.000-2.350) |
|  | A/A | 1.440 (0.000-3.610) | 0.650 (0.000-2.300) |
|  | *P* | 0.741 | 0.623 |
| rs10208769 | A/A | 1.800 (0.400-3.725) | 0.700 (0.000-2.200) |
|  | A/T | 1.610 (0.020-2.875) | 1.000 (0.000-2.600) |
|  | T/T | 2.000 (0.400-2.930) | 0.900 (0.100-2.245) |
|  | *P* | 0.317 | 0.272 |
| rs2123111 | G/G | 1.800 (0.180-3.535) | 0.700 (0.000-2.100) |
|  | G/A | 1.626 (0.270-2.955) | 0.950 (0.000-2.600) |
|  | A/A | 2.000 (0.460-2.948) | 0.900 (0.200-2.395) |
|  | *P* | 0.643 | 0.086 |
| rs6545910 | C/C | 1.920 (0.400-3.510) | 0.900 (0.000-2.400) |
|  | C/T | 1.600 (0.000-2.835) | 0.550 (0.000-2.175) |
|  | T/T | 1.800 (0.000-3.000) | 1.400 (0.000-3.600) |
|  | *P* | 0.206 | 0.082 |
| rs6748320 | G/G | 1.700 (0.400-3.280) | 0.900 (0.000-2.200) |
|  | G/A | 1.700 (0.000-3.375) | 0.700 (0.000-2.400) |
|  | A/A | 2.205 (0.742-3.413) | 1.000 (0.000-2.675) |
|  | *P* | 0.459 | 0.592 |
| rs3736598 | G/G | 1.600 (0.385-3.025) | 0.900 (0.000-2.050) |
|  | G/A | 1.710 (0.020-3.400) | 0.800 (0.000-2.450) |
|  | A/A | 2.210 (0.695-3.425) | 1.000 (0.000-2.700) |
|  | *P* | 0.410 | 0.528 |
| rs777585 | T/T | 2.000 (0.200-3.400) | 0.900 (0.000-2.345) |
|  | T/C | 1.770 (0.400-3.150) | 0.900 (0.000-2.500) |
|  | C/C | 1.250 (0.000-3.200) | 0.400 (0.000-2.100) |
|  | *P* | 0.521 | 0.631 |
| rs3811616 | A/A | 1.800 (0.400-3.200) | 0.870 (0.000-2.300) |
|  | A/G | 1.600 (0.000-3.400) | 1.000 (0.000-2.575) |
|  | G/G | 1.626 (0.200-3.210) | 0.200 (0.000-2.000) |
|  | *P* | 0.974 | 0.109 |
| rs1729674 | T/T | 1.780 (0.200-3.450) | 0.700 (0.000-2.100) |
|  | T/G | 1.600 (0.008-2.900) | 0.900 (0.000-2.725) |
|  | G/G | 2.140 (0.795-3.415) | 1.000 (0.300-2.300) |
|  | *P* | 0.246 | 0.073 |
| rs55785307 | C/C | 1.685 (0.400-3.380) | 0.900 (0.000-2.345) |
|  | C/G | 1.825 (0.000-3.162) | 0.900 (0.000-2.500) |
|  | G/G | 1.200 (0.300-3.392) | 0.785 (0.000-2.500) |
|  | *P* | 0.929 | 0.923 |
| rs1177284 | G/G | 1.800 (0.220-3.600) | 0.900 (0.000-2.238) |
|  | G/A | 1.600 (0.000-2.958) | 0.750 (0.000-2.500) |
|  | A/A | 2.050 (0.465-3.510) | 1.000 (0.200-2.425) |
|  | *P* | 0.297 | 0.184 |
| rs10865331 | G/G | 1.775 (0.305-3.175) | 0.900 (0.000-2.200) |
|  | G/A | 1.729 (0.400-3.210) | 0.900 (0.000-2.625) |
|  | A/A | 1.710 (0.000-3.750) | 0.900 (0.000-2.100) |
|  | *P* | 0.819 | 0.678 |

BASDAI, Bath Ankylosing Spondylitis Disease Activity Index; BASFI, Bath Ankylosing Spondylitis Functional Index;

P_25_, lower quartile; P_75_, upper quartile.
